# Distribution of Human Papillomavirus Genotypes among HIV-Positive and HIV-Negative Women in Cape Town, South Africa

**DOI:** 10.3389/fonc.2014.00048

**Published:** 2014-03-14

**Authors:** Alicia C. McDonald, Ana I. Tergas, Louise Kuhn, Lynette Denny, Thomas C. Wright

**Affiliations:** ^1^Department of Population Health, Hofstra North Shore Long Island Jewish School of Medicine, Great Neck, NY, USA; ^2^Feinstein Institute for Medical Research, North Shore Long Island Jewish Health System, Manhasset, NY, USA; ^3^Department of Epidemiology, Mailman School of Public Health, Columbia University, New York, NY, USA; ^4^Department of Obstetrics and Gynecology, College of Physicians and Surgeons, Columbia University, New York, NY, USA; ^5^Gertrude H. Sergievsky Center, College of Physicians and Surgeons, Columbia University, New York, NY, USA; ^6^Department of Obstetrics and Gynaecology, Institute of Infectious Diseases and Molecular Medicine, University of Cape Town, Cape Town, South Africa; ^7^Department of Pathology, College of Physicians and Surgeons, Columbia University, New York, NY, USA

**Keywords:** HIV-infections, HPV, genotype, HPV vaccine, cervical cancer screening

## Abstract

**Objective:** HIV-positive women are known to be at high-risk of human papillomavirus (HPV) infection and its associated cervical pathology. Here, we describe the prevalence and distribution of HPV genotypes among HIV-positive and -negative women in South Africa, with and without cervical intraepithelial neoplasia (CIN).

**Methods:** We report data on 1,371 HIV-positive women and 8,050 HIV-negative women, aged 17–65 years, recruited into three sequential studies in Cape Town, South Africa, conducted among women who had no history of cervical cancer screening recruited from the general population. All women were tested for HIV. Cervical samples were tested for high-risk HPV DNA (Hybrid Capture 2) with positive samples tested to determine the specific genotype (Line Blot). CIN status was determined based on colposcopy and biopsy.

**Results:** The HPV prevalence was higher among HIV-positive women (52.4%) than among HIV-negative women (20.8%) overall and in all age groups. Younger women, aged 17–19 years, had the highest HPV prevalence regardless of HIV status. HIV-positive women were more likely to have CIN 2 or 3 than HIV-negative women. HPV 16, 35, and 58 were the most common high-risk HPV types with no major differences in the type distribution by HIV status. HPV 18 was more common in older HIV-positive women (40–65 years) with no or low grade disease, but less common in younger women (17–29 years) with CIN 2 or 3 compared to HIV-negative counterparts (*p* < 0.03). Infections with multiple high-risk HPV types were more common in HIV-positive than HIV-negative women, controlling for age and cervical disease status.

**Conclusion:** HIV-positive women were more likely to have high-risk HPV than HIV-negative women; but, among those with HPV, the distribution of HPV types was similar by HIV status. Screening strategies incorporating HPV genotyping and vaccination should be effective in preventing cervical cancer in both HIV-positive and -negative women living in sub-Saharan Africa.

## Introduction

Persistent infection with one of the 13 high oncogenic risk human papillomavirus genotypes (hrHPV) is now firmly established as the cause of almost all cervical cancers, as well as a significant proportion of other anogenital cancers and head and neck cancers in men and women (1). Both hrHPV and HIV are sexually transmitted infectious agents and infection with either one of the two viruses may facilitate transmission of the other (2, 3). Numerous studies have shown that HIV-positive women have a higher prevalence of hrHPV infections than HIV-negative women (4–6). Moreover, hrHPV infections are more likely to be persistent in HIV-positive women; and, a very high prevalence of high-grade cervical cancer precursors referred to as cervical intraepithelial neoplasia grades 2 and 3 (CIN 2 or 3) are found in HIV-positive women (2–4, 6–8). Tumor registries and prospective follow-up studies from the United States and Europe have shown the incidence of invasive cervical cancer to be higher in HIV-positive women (9–13).

The most important determinant of whether or not a hrHPV infection will persist and progress to a CIN 2 or 3 lesion or an invasive cervical cancer is the specific genotype of hrHPV with which an individual is infected. For example, HPV 16 is associated with especially elevated rates of persistence and progression than other hrHPV genotypes (14, 15). Some evidence suggests that HIV-positive women are infected with a broader range of hrHPV genotypes than HIV-negative women (16–18). If true, this would have important implications for the effectiveness of both HPV vaccination and the use of hrHPV genotyping assays for screening HIV-positive women. Relatively few large studies have directly compared the distribution of hrHPV genotypes in HIV-positive and -negative women of known cervical disease status; and, the evaluation of HPV testing as a screening strategy among HIV-positive women is warranted. Such studies are especially needed for sub-Saharan African populations where the prevalence of both HIV-infection and HPV-associated cervical cancer is particularly high. There are approximately 23.5 million adults and children living with HIV in sub-Saharan Africa; and, the incidence and mortality from cervical cancer among women living in sub-Saharan Africa are among the highest in the world with 70,000 new cases, annually (19–21).

We have conducted several cervical cancer screening trials in Cape Town, South Africa in which both HIV-positive and -negative women were enrolled; and, disease endpoints were rigorously ascertained using colposcopy and cervical biopsy. In a previous study, we reported that HPV 16 and 35 were the most common high-risk types among HIV-negative women (22); however, we did not examine high-risk HPV genotypes among HIV-positive women. In this study, we compare the distribution and prevalence of specific hrHPV genotypes in HIV-positive and -negative women of known cervical disease status who were enrolled in either one of three sequential cervical cancer screening studies that we conducted in South Africa. We also compare the performance of hrHPV testing as a screening test for cervical disease in HIV-positive and -negative women.

## Materials and Methods

### Study population

We report a secondary analysis of data collected as part of three sequential studies that recruited healthy women from the general population at three clinical sites in the community of Khayelitsha, Cape Town, South Africa. All three studies included women who were not pregnant at the time of enrollment, had never been screened or treated for cervical cancer, and had not undergone a hysterectomy. Women were recruited through community outreach activities, including handing out fliers at bus and train stations, meeting with church and women’s groups in the area, and advertising on radio programs. All women provided written informed consent; and, the protocols were approved by the Institutional Review Boards of Columbia University, New York, NY, USA and the University of Cape Town, Cape Town, South Africa.

In Cohort 1, 191 HIV-positive and 2,505 HIV-negative women, aged 35–65 years, were enrolled between January 1998 and November 1999 into a study evaluating the performance of different tests for cervical cancer screening (23). In Cohort 2, 841 HIV-positive and 5,708 HIV-negative women, aged 35–65 years, were enrolled between June 2000 and December 2002 and were followed for 36 months in a trial examining the safety and efficacy of two screen-and-treat approaches for cervical cancer prevention (24). For the analyses presented here, only women randomized to the control group (284 HIV-positive and 1,881 HIV-negative) or to the screen-and-treat arm utilizing HPV testing (286 HIV-positive and 1,874 HIV-negative) were included due to the availability of HPV typing data, giving a total of 4,325 women in Cohort 2. In Cohort 3, 733 HIV-positive and 2,265 HIV-negative, aged 17–34 years, were enrolled in a study between July 2004 and June 2006 examining HPV prevalence among younger women. There were no duplicate women in the three cohorts to our knowledge. For the current analysis, we further restricted the study population by excluding women with no known definitive cervical disease status (*n* = 574) and women with invasive cervical cancer who were described separately in this study (*n* = 24), resulting in a final sample size of 1,371 HIV-positive and 8,050 HIV-negative women (190 HIV-positive and 2,485 HIV-negative in Cohort 1, 494 HIV-positive and 3,353 HIV-negative in Cohort 2, and 687 HIV-positive and 2,212 HIV-negative in Cohort 3).

### Study procedures

In all three studies, a short risk factor questionnaire was conducted at baseline and blood was collected for HIV testing. A gynecologic examination was conducted in which cervical samples were obtained, including a sample collected from the exo- and endo-cervix using a plastic spatula and cytobrush and placed into liquid-based cytology (LBC) medium (ThinPrep PreservCyte, Hologics, Marlborough, MA, USA), which was used for HPV testing. Pap smears were evaluated at the University of Cape Town Cytopathology Laboratory, Health Networks Laboratory, Allentown, PA, USA or Columbia University, New York, NY, USA and were classified using the Bethesda System.

### Laboratory procedures

Hybrid capture 2 (HC2) DNA assay (Qiagen, Germantown, MD, USA) was used to test cervical samples for HPV DNA types 16, 18, 31, 33, 35, 39, 45, 51, 52, 56, 58, 59, and 68 (24). HPV DNA positivity was based on a cut-off of relative light units (RLU) >1 time the positive control. All testing was done at the University of Cape Town when the samples were collected. Aliquots of the LBC samples were stored at −30°C and shipped to Columbia University for future testing.

Stored cervical samples from all women who were HC2 positive were sought for determination of the specific HPV genotype present. Of 9,421 women in the three cohorts, 2,389 samples tested HPV positive by HC2 and 2,354 (98.5%) could be located and further tested to determine the specific high-risk HPV genotype present. DNA was isolated from 200 μl of the LBC specimen (Qiagen, Chatsworth, CA, USA) and purified DNA was analyzed for individual HPV genotypes using a prototype polymerase chain reaction (PCR)-based line blot assay (kindly provided by Dr. Janet Kornegay, Roche Molecular Diagnostics, Alameda, CA, USA) that uses the PGMY09/11 consensus primers (25). If a high-risk HPV genotype was not identified using the prototype line blot assay, DNA was re-isolated and re-analyzed for individual HPV genotypes using the PCR-based, Linear Array HPV Typing Assay (Roche Molecular Diagnostics, Alameda, CA, USA) (25). HPV typing was done in batches at Columbia University, Department of Pathology, after the completion of each study following the manufacturers’ instructions and reagents provided at the time of each batch (25).

### Disease status determination

To meet the objectives of each study, slightly different protocols were followed in each cohort to determine final disease status [within normal limits (WNL), CIN (CIN) grade 1 (CIN 1), CIN grade 2 (CIN 2), CIN grade 3 (CIN 3)]. In Cohort 1, all women who had positive results on one or more of four independent screening tests were referred for colposcopy 2–6 days after the enrollment visit. The four screening tests were HPV DNA testing using HC2, visual inspection with acetic acid, cytology (ASCUS and above were referred), and expert cervicography (23). Approximately half of the participants had one or more of the four screening tests classified as positive and underwent colposcopy. In Cohort 2, colposcopy was performed on all women at 6 and 12 months after enrollment (24). Samples for HPV DNA testing were collected at the time of enrollment. In Cohort 3, all women underwent colposcopy at their enrollment examination. Women not found to have biopsy-confirmed CIN 2 or greater at the initial colposcopy who were HC2 positive, had cytology results of ≥ASCUS, or who had biopsy-confirmed CIN 1 lesions underwent a second colposcopy 12 weeks after enrollment. Thus, for both Cohorts 2 and 3, all subjects underwent at least one or more colposcopy examinations. Due to the fact that no cervical disease was diagnosed in Cohort 2 or 3 among women who had both negative HPV and cytology results, we can confirm that minimal verification bias exists in Cohort 1. In all studies, colposcopy was conducted by clinicians specifically trained in colposcopy and according to a standard protocol. All abnormal areas were biopsied and endocervical curettage specimens were collected. Biopsy and endocervical curettage specimens were evaluated by two pathologists at Columbia University. Inconsistent diagnoses were adjudicated in a microscopic conference; and, the final disease status was based on the highest grade adjudicated pathology diagnosis.

### Data analysis

Proportions were calculated and compared using Pearson’s chi-square test or Fisher’s exact test where numbers were small. Mann–Whitney *U* test was used to examine whether there were differences in median values for continuous variables between HIV status groups. HPV prevalence (HC2 DNA positivity) was calculated as the number of positive women divided by the total number of women. The distribution of HPV genotypes was calculated as the number of women with a specific high-risk HPV type divided by the number of detected high-risk HPV types/infections found among these women and also by dividing by the number of women with any high-risk type. Analysis was conducted using SAS statistical software (Cary, NC, USA).

## Results

### Study population

**Figure 1:**
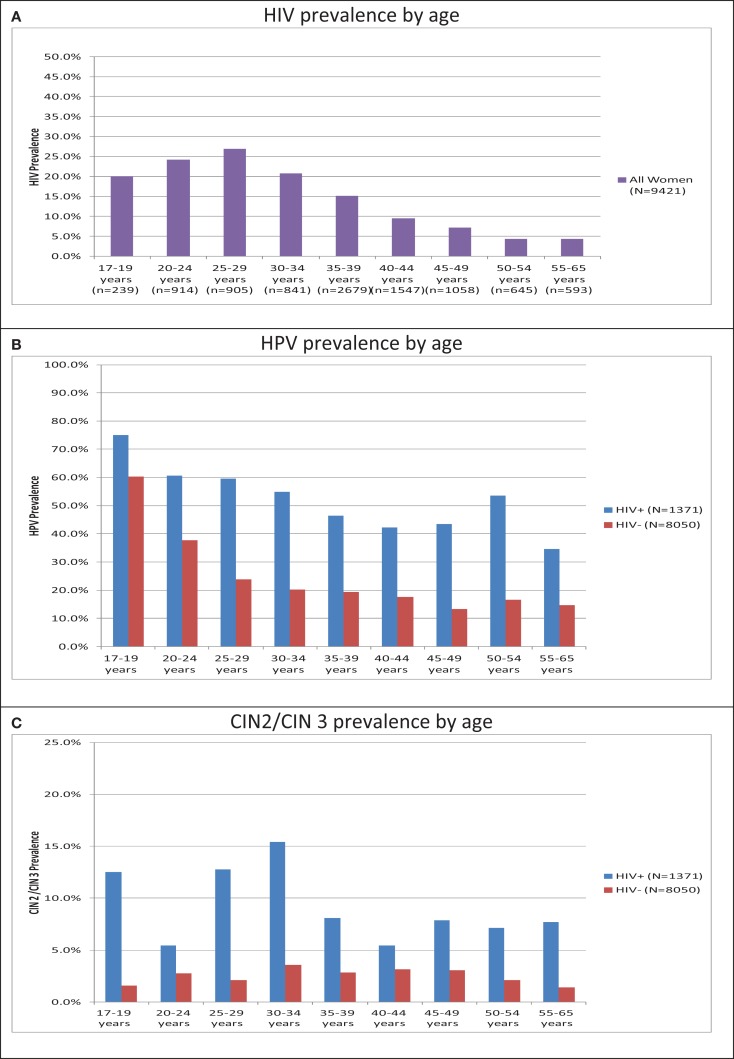
**Age-specific HIV, HPV, and cervical disease prevalence in 9,421 women recruited in Cape Town, South Africa**. **(A)** HIV prevalence by age. **(B)** HPV prevalence by age. **(C)** CIN2/CIN 3 prevalence by age. Note: CIN, cervical intraepithelial neoplasia; HPV, human papillomavirus. See Supplementary Material for data.

Of the 9,421 women included, 14.6% (*n* = 1,371) were HIV-positive. The prevalence of HIV increased from 20.1% in women, aged 17–19 years, to a peak of 26.9% in women, aged 25–29 years, and declined thereafter to 4.4% in women, aged 55–65 years (Figure 1A). HIV-positive women were quite distinct from HIV-negative women in demographic, HPV, and CIN parameters (Table 1). HIV-positive women were younger, less educated, less likely to be employed, and less likely to be married than HIV-negative women (*p* < 0.0001). HIV-positive women had an earlier age of first sexual intercourse and were more likely to be treated for a sexually transmitted disease, but were more likely to use condoms than HIV-negative women (*p* < 0.0001).

**Table 1:** **Characteristics of HIV-positive and HIV-negative women who participated in cervical cancer screening studies in Khayelitsha, South Africa**.

	Overall (*N* = 9,421)	HIV-positive (*N* = 1,371)	HIV-negative (*N* = 8,050)	*p*-Value
Median age (25th and 75th percentile)	37 years (31–44 years)	34 years (26–38 years)	38 years (33–45 years)	<0.0001
% ≤10 years of education	80.5% (7,579/9,421)	75.7% (1,038/1,371)	81.3% (6,541/8,050)	<0.0001
% Currently employed	30.6% (2,878/9,401)	26.2% (359/1,370)	31.4% (2,519/8,031)	0.0001
% Smoker	5.5% (521/9,417)	6.5% (89/1,370)	5.4% (432/8,047)	0.0914
% Married	44.0% (4,148/9,421)	26.9% (369/1,371)	46.9% (3,779/8,050)	<0.0001
Median age of first sexual intercourse (*n*; range)	17 years (9,416; 6–39 years)	16 years (1,369; 7–30 years)	17 years (8,047; 6–39 years)	<0.0001
% Ever treated for STD	9.1% (858/9,403)	14.6% (200/1,371)	8.2% (658/8,032)	<0.0001
% Ever used condoms	27.2% (2,288/8,404)	40.6% (524/1,290)	24.8% (1,764/7,114)	<0.0001
% Disease status
WNL	91.3% (8,601/9,421)	75.3% (1,032/1,371)	94.0% (7,569/8,050)	<0.0001^a^
CIN 1	5.0% (473/9,421)	15.5% (212/1,371)	3.2% (261/8,050)	
CIN 2	2.5% (231/9,421)	7.2% (99/1,371)	1.6% (132/8,050)	
CIN 3	1.2% (116/9,421)	2.0% (28/1,371)	1.1% (88/8,050)	
% HPV DNA positive (HPV prevalence)	25.4% (2,389/9,421)	52.4% (719/1,371)	20.7% (1,670/8,050)	<0.0001
% Disease status among HPV positives
WNL	70.3% (1,679/2,389)	55.2% (397/719)	76.8% (1,282/1,670)	–
CIN 1	16.6% (396/2,389)	27.2% (196/719)	12.0% (200/1,670)	<0.0001^b^
CIN 2	8.7% (207/2,389)	13.8% (99/719)	6.5% (108/1,670)	<0.0001^b^
CIN 3	4.5% (107/2,389)	3.8% (27/719)	4.8% (80/1,670)	0.7081^b^

STD, sexually transmitted disease; HPV, human papillomavirus; WNL, within normal limits; CIN, cervical intraepithelial neoplasia;

^a^*p*-value for trend;

*^b^*p*-value of the comparison of cervical disease with WNL between HIV-positive and -negative women*.

### High-risk HPV prevalence

The prevalence of high-risk HPV DNA detected by HC2 was higher among HIV-positive than HIV-negative women within all age groups. In the youngest age group (17–19 years), this difference was the least marked: 75% of HIV-positive women were HPV DNA positive compared to 60.2% of HIV-negative women (*p* = 0.06). In older age groups, the prevalence of HPV DNA in HIV-positive women was consistently more than twice that observed in HIV-negative women. For example, the HPV prevalence in HIV-positive vs. -negative women, aged 25–29 years and 35–39 years, was 59.7% vs. 23.9% (*p* < 0.0001) and 46.4% vs. 19.3% (*p* < 0.0001), respectively. For both HIV-positive and -negative women, the prevalence of HPV declined steadily from the 17- to 19-year age category to its lowest level among women in their 40’s (Figure 1B). In HIV-positive women, there was a slight upward trend among women, aged 50–54 years; however, there were only 28 women in this group.

### Cervical intraepithelial neoplasia grades 2 and 3

The prevalence of CIN 2 and CIN 3 was significantly higher among HIV-positive women. The prevalence of CIN 2 was 7.2% among HIV-positive vs. 1.6% among HIV-negative women (*p* < 0.001); and, the prevalence of CIN 3 was 2.0% among HIV-positive vs. 1.1% among HIV-negative women (*p* < 0.001) (Table 1). As seen in Figure 1C, this higher prevalence of CIN 2 or 3 in HIV-positive vs. HIV-negative women was consistent across all age categories. The differences in CIN 2 or 3 prevalence by HIV status were particularly marked among younger women (<35 years of age) but remained statistically significant in the 35- to 39-year and 45- to 49-year age categories.

Restricting to those women who were HPV DNA positive, the prevalence of CIN 1 and CIN 2, respectively, were higher among HIV-positive (27.2% and 13.8%) than among HIV-negative (12.0% and 6.5%) women. However, in those who were HPV DNA positive, the prevalence of CIN 3 was not significantly different by HIV status: 3.8% HIV-positive vs. 4.8% HIV-negative women (Table 1).

### Performance characteristics of HPV DNA testing

HIV status did not compromise the sensitivity of HPV testing to detect any of the grades of disease. Rather, there was a greater sensitivity of HPV testing to detect CIN among HIV-positive women, although the sensitivity was high (>80%) in HIV-negative women as well (Table 2). Specificity of HPV DNA testing was significantly lower among HIV-positive compared to HIV-negative women. Specificity was especially poor for HIV-positive women if CIN 1 was considered to be absence of disease. Inclusion or exclusion of CIN 1 did not influence the specificity estimates as much for the HIV-negative women. Positive predictive value was consistently higher for HIV-positive than HIV-negative women except for the detection of CIN 3, as mentioned above. Negative predictive value was very high in both groups.

**Table 2:** **Performance characteristics of HPV DNA testing using HC2 to detect different grades of disease among 1,371 HIV-positive and 8,050 HIV-negative women**.

	HIV-positive women	HIV-negative women	*p*-Value
Sensitivity to detect CIN 3^a^	96.4% (27/28)	90.9% (80/88)	0.6852
Sensitivity to detect CIN 2^a^	100% (99/99)	81.8% (108/132)	<0.0001
Sensitivity to detect CIN 1^a^	92.5% (196/212)	76.6% (200/261)	<0.0001
Sensitivity to detect CIN 2 or 3^a^	99.2% (126/127)	85.5% (188/220)	<0.0001
Sensitivity to detect CIN 1, 2, or 3^a^	95.0% (322/339)	80.7% (388/481)	<0.0001
Specificity (WNL)^b^	61.5% (635/1,032)	83.1% (6,287/7,569)	<0.0001
Specificity (WNL or CIN 1)^c^	52.3% (651/1,244)	81.1% (6,348/7,830)	<0.0001
Positive predictive value (CIN 2 or 3)^c^	17.5% (126/719)	11.3% (188/1,670)	<0.0001
Positive predictive value (CIN 1, 2, or 3)^a^	44.8% (322/719)	23.2% (388/1,670)	<0.0001
Negative predictive value (WNL)^b^	97.4% (635/652)	98.5% (6,287/6,380)	0.0242
Negative predictive value (WNL or CIN 1)^c^	99.8% (651/652)	99.5% (6,348/6,380)	0.3619

HPV, human papillomavirus; WNL, within normal limits; CIN, cervical intraepithelial neoplasia;

^a^*p*-value of the comparison between cervical disease(s) and WNL;

^b^*p*-value of the comparison between WNL and CIN 1/2/3;

*^c^*p*-value of the comparison between WNL/CIN 1 and CIN 2/3*.

### Distribution of specific high-risk HPV genotypes

Of 2,354 women who tested HC2 positive and whose samples could be located and tested to determine the specific high-risk HPV genotype present, one or more high-risk HPV genotypes were detected in 1,848 women [609/712 (85.5%) HIV-positive vs. 1,239/1,642 (75.5%) HIV-negative women]. This detection was higher among women who had CIN: 1,217 [315/395 (79.8%) HIV-positive vs. 902/1,261 (71.5%) HIV-negative] women with WNL, 349 [177/193 (91.7%) HIV-positive vs. 172/198 (86.9%) HIV-negative] women with CIN 1, 183 [90/97 (92.8%) HIV-positive vs. 93/103 (90.3%) HIV-negative] women with CIN 2, and 99 [27/27 (100%) HIV-positive vs. 72/80 (90%) HIV-negative] women with CIN 3.

Overall, the most common high-risk HPV genotypes among both HIV-positive and HIV-negative women were HPV 16, 35, and 58 (in descending order). There were only slight differences in the high-risk HPV type distribution when the number of women with high-risk HPV types was used as the denominator (Table 3). Women with CIN 3 were more likely to have HPV 16 than women with CIN 2 or less; this pattern was consistent across HIV-positive and -negative women. The most common high-risk HPV types in women with CIN 3 were the following: HPV 16, 33 and 35 (tied), and 58 in HIV-positive women, and HPV 16, 35, and 45 in HIV-negative women (in descending order). HIV-positive women had more HPV 18 than HIV-negative women if they had no cervical disease; they had more HPV 39 and 58 than HIV-negative women if they had CIN 1; they had more HPV 68 than HIV-negative women if they had CIN 2; and, they had more HPV 39 and HPV 68 than HIV-negative women if they had CIN 3 (*p* < 0.05 for each) (Table 3). When the number of high-risk HPV infections was used as the denominator (instead of the number of women with high-risk types), there were no differences in the distribution of hrHPV types by HIV status.

**Table 3:** **Distribution of specific high-risk HPV genotypes among 609 HIV-positive women and 1,239 HIV-negative women with a high-risk genotype detected by PCR**.

	WNL		CIN 1		CIN 2		CIN 3	
	HIV- positive	HIV- negative	HIV- positive	HIV- negative	HIV- positive	HIV- negative	HIV- positive	HIV- negative
# Of women	1,032	7,569	212	261	99	132	28	88
# Of HC2 positive women	397	1,282	196	200	99	108	27	80
# Of PCR, HC2 positive women	395	1,261	193	198	97	103	27	80
# Women with HR types	315	902	177	172	90	93	27	72
# Of HR infection types	471	1,164	311	247	155	126	54	100
*N* (%)*								
Type 16	45 (14.3)	146 (16.2)	33 (18.6)	23 (13.4)	20 (22.2)	21 (22.6)	14 (51.9)	29 (40.3)
Type 18	46 (14.6)	85 (9.4)*	26 (14.7)	22 (12.8)	9 (10.0)	9 (9.7)	4 (14.8)	8 (11.1)
Type 31	24 (7.6)	72 (8.0)	16 (9.0)	17 (9.9)	14 (15.6)	8 (8.6)	2 (7.4)	9 (12.5)
Type 33	29 (9.2)	67 (7.4)	13 (7.3)	15 (8.7)	10 (11.1)	12 (12.9)	7 (25.9)	9 (12.5)
Type 35	54 (17.1)	151 (16.7)	33 (18.6)	41 (23.8)	23 (25.6)	29 (31.2)	7 (25.9)	14 (19.4)
Type 39	25 (7.9)	51 (5.7)	17 (9.6)	7 (4.1)*	5 (5.6)	1 (1.1)	4 (14.8)	2 (2.8)*
Type 45	46 (14.6)	106 (11.8)	23 (13.0)	19 (11.1)	7 (7.8)	7 (7.5)	2 (7.4)	12 (16.7)
Type 51	29 (9.2)	75 (8.3)	30 (17.0)	23 (13.4)	11 (12.2)	5 (5.4)	0 (0)	1 (1.4)
Type 52	37 (11.8)	90 (10.0)	26 (14.7)	21 (12.2)	9 (10.0)	12 (12.9)	2 (7.4)	3 (4.2)
Type 56	22 (7.0)	56 (6.2)	19 (10.7)	15 (8.7)	8 (8.9)	3 (3.2)	2 (7.4)	1 (1.4)
Type 58	50 (15.9)	115 (12.8)	31 (17.5)	14 (8.1)*	22 (24.4)	13 (14.0)	5 (18.5)	9 (12.5)
Type 59	22 (7.0)	65 (7.2)	17 (9.6)	14 (8.1)	5 (5.6)	2 (2.2)	1 (3.7)	1 (1.4)
Type 68	42 (13.3)	85 (9.4)	27 (15.3)	16 (9.3)	12 (13.3)	4 (4.3)*	4 (14.8)	2 (2.8)*
Types 16/18	85 (27.0)	229 (25.4)	56 (31.6)	43 (25.0)	28 (31.1)	29 (31.28)	18 (66.7)	35 (48.6)

HPV, human papillomavirus; WNL, within normal limits; CIN, cervical intraepithelial neoplasia; HR, high-risk: *% is calculated using the number of women with any high-risk HPV type as the denominator, *p-values <0.05.

**Figure 2:**
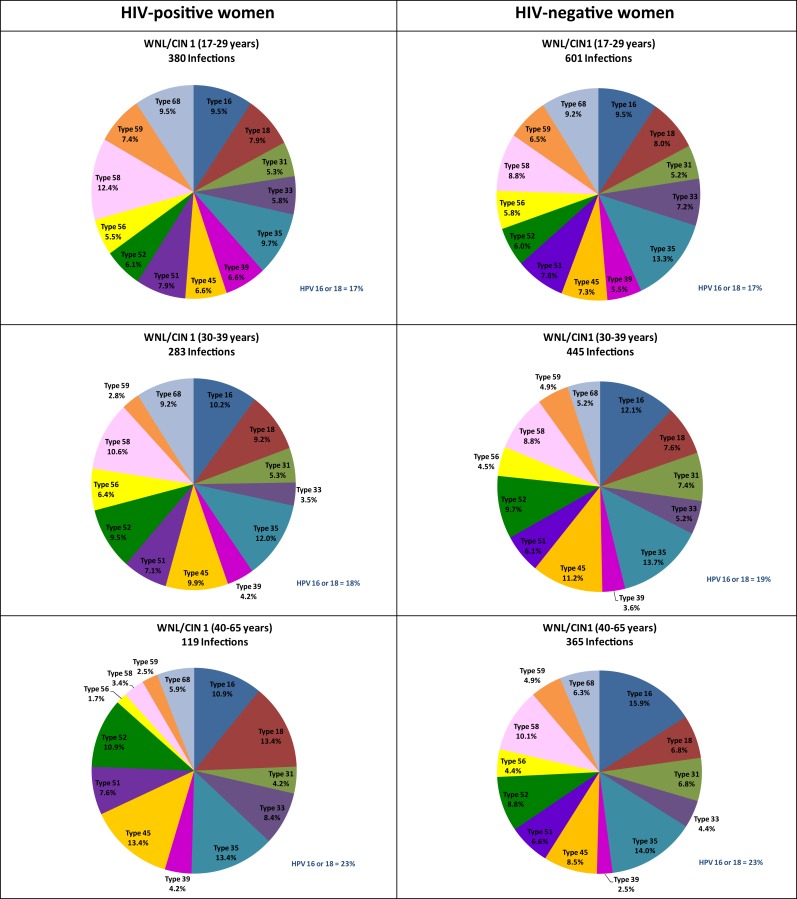
**Age-specific distribution of high-risk HPV genotypes of 782 infections among 492 HIV-positive women and 1,411 infections among 1,074 HIV-negative women with within normal limits or cervical intraepithelial neoplasia grade 1 only**. Note: WNL, within normal limits; CIN, cervical intraepithelial neoplasia; HR, high-risk; HPV, human papillomavirus. See Supplementary Material for data.

**Figure 3:**
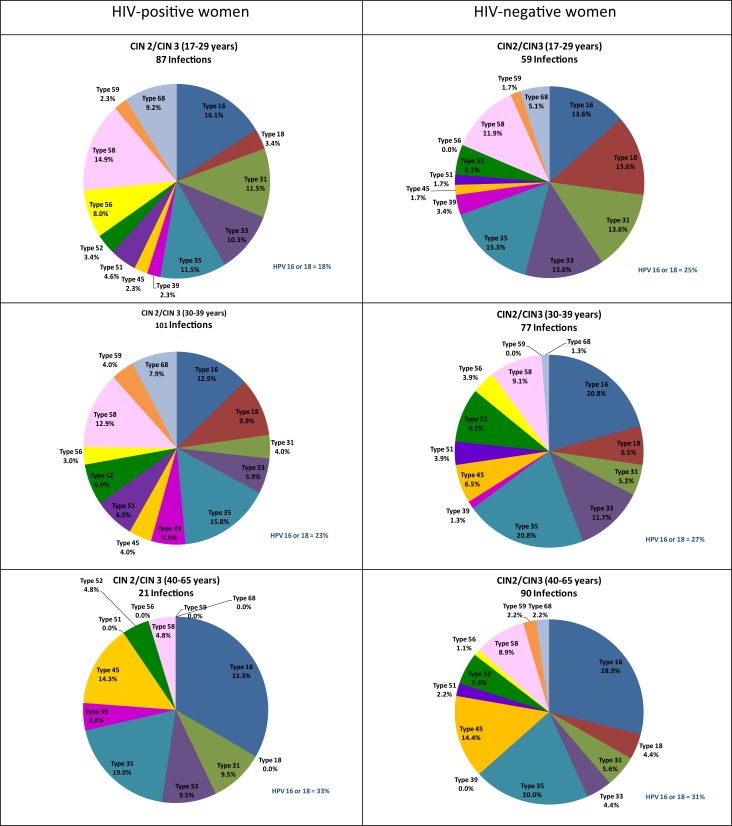
**Age-specific distribution of high-risk HPV genotypes of 209 infections among 117 HIV-positive women and 226 infections among 165 HIV-negative women with cervical intraepithelial neoplasia grade 2 or 3**. Note: WNL, within normal limits; CIN, cervical intraepithelial neoplasia; HR, high-risk; HPV, human papillomavirus. See Supplementary Material for data.

Further stratification by age as well as CIN status revealed some differences in the distribution of HPV types by HIV status even when using the number of high-risk infections as the denominator. Among women, aged 40–65 years, with WNL or CIN 1, HIV-positive women had a significantly greater representation of HPV 18 and a significantly lesser representation of HPV 58 than HIV-negative women of similar age and cervical disease status. Among women, aged 30–39 years, with WNL or CIN 1, HIV-positive women had a significantly greater representation of HPV 68 than HIV-negative women of similar age and cervical disease status (Figure 2). Among women, aged 17–29 years, with CIN 2 or 3, HIV-positive women had a significantly greater representation of HPV 56 and a significantly lesser representation of HPV 18 than HIV-negative women (Figure 3).

### Multiple HPV genotypes

Table 4 shows the frequency of multiple HPV genotypes by age, cervical disease, and HIV status among women with at least one high-risk HPV genotype detected. In women with WNL or CIN 1, HIV-positive women had significantly more HPV infections with multiple high-risk types than HIV-negative women across all age groups. In women with CIN 2 or 3, the differences by HIV status were weaker than the differences observed in women with WNL or CIN 1. HIV-positive women with CIN 2 or 3 had more multiple high-risk HPV types compared to HIV-negative women; however, only HIV-positive women, aged 30–39 years, had significantly more multiple high-risk HPV types than their HIV-negative counterparts. The youngest age group, 17–29 years, had more HPV infection with multiple high-risk types compared to older age groups, regardless of cervical disease and HIV status.

**Table 4:** **Multiple high-risk types in HIV-positive (*N* = 609) and -negative (*N* = 1,239) women by age and disease status**.

	HIV-positive				HIV-negative				*p*-Value (HIV+ vs. HIV−)
	*N* with at least 1 HR type	Median (range)	*N* with 1 HR type (%)	*N* with 2+ types (%)	*N* with at least 1 HR type	Median (range)	*N* with 1 HR type (%)	*N* with 2+ types (%)	
**WNL/CIN 1**									
17–29 years	221	1 (1–5)	120 (54.3)	101 (45.7)	403	1 (1–6)	264 (65.5)	139 (34.5)	0.0059
30–39 years	184	1 (1–7)	120 (65.2)	64 (34.8)	358	1 (1–4)	288 (80.5)	70 (19.6)	<0.0001
40–65 years	87	1 (1–4)	64 (73.6)	23 (26.4)	313	1 (1–3)	264 (84.4)	49 (15.7)	0.0206
**CIN 2/CIN 3**									
17–29 years	46	2 (1–4)	20 (43.5)	26 (56.5)	32	1 (1–6)	17 (53.1)	15 (46.9)	0.4013
30–39 years	56	2 (1–4)	26 (46.4)	30 (53.6)	62	1 (1–3)	49 (79.0)	13 (21.0)	0.0002
40–65 years	15	1 (1–3)	10 (66.7)	5 (33.3)	71	1 (1–4)	56 (78.9)	15 (21.1)	0.3093
Total population	609	1 (1–7)	360 (59.1)	249 (40.9)	1,239	1 (1–6)	938 (75.7)	301 (24.3)	<0.0001

*WNL, within normal limits; CIN, cervical intraepithelial neoplasia; HR, high-risk; *N*, number*.

### Cervical cancer

Twenty-four women with cancer were identified (1 HIV-positive woman and 23 HIV-negative women). The one HIV-positive woman was HC2 negative and had no hrHPV types identified on PCR. Among 23 HIV-negative women with cervical cancer, 19 (82.6%) were HC2 positive and 18 of these had a hrHPV type detected by PCR. Fourteen of 18 (77.8%) had either HPV 16 (*n* = 10) or HPV 18 (*n* = 4); two (11.1%) women had HPV 45; two (11.1%) women had HPV 58; and, one (5.6%) woman had HPV 68.

## Discussion

To our knowledge, our study is the largest one to date to compare the distribution and prevalence of specific hrHPV genotypes in sub-Saharan African HIV-positive and -negative women of known cervical disease status. Our study confirms a higher overall prevalence of hrHPV infection, more cervical disease, and a greater proportion of infections with multiple genotypes of hrHPV in HIV-positive women compared to HIV-negative women. These findings, although limited by the lack of detailed information on the severity of HIV disease, are consistent with previous studies reporting higher HPV prevalence (26–30), more cervical abnormalities (26, 29, 31), and more multiple high-risk HPV infections (6, 8, 27–30, 32–34) in HIV-positive compared to HIV-negative women. As shown in a large meta-analysis of hrHPV prevalence studies in developing countries (35), the prevalence of hrHPV infection in both groups of women was highest in young women and steadily declined until age of 45–49 years, increasing somewhat in women, aged 50–54 years. Across almost all age groups, the hrHPV prevalence in HIV-positive women was more than twice that observed in HIV-negative women. Whether the higher age-stratified prevalences among HIV-positive women are due to greater HPV persistence/reactivation, behavior differences, or consequences of HIV infection and concomitant immunosuppression, these factors can not be resolved with our data (36–38). Nevertheless, the high HPV prevalences translate into high rates of cervical precursor lesions, making HIV-positive women a priority for public health interventions.

Only minor differences were observed in the relative distribution of hrHPV genotypes in HIV-positive women compared to HIV-negative women when stratified by biopsy-confirmed cervical disease status. Since the histological diagnosis of CIN 1 is poorly reproducible, we combined no cervical disease together with CIN 1 into a single category (39). Among the 593 HIV-positive and 1,482 HIV-negative women who were hrHPV-positive and had no cervical disease or biopsy-confirmed CIN 1, not a single hrHPV genotype had a significantly different prevalence based on HIV serostatus. The most common hrHPV genotype was HPV 35, closely followed by HPV 16 and 58. Because the hrHPV prevalence is so strongly affected by age, we also performed age-stratified analyses. Although a few statistically significant differences by HIV status were observed, the differences are likely explained by chance and would not persist if adjustments were made for multiple comparisons. Importantly for HPV vaccine effectiveness, the combined prevalence of HPV 16 and 18 was almost identical between HIV-positive and -negative women in all age groups. Similarly, among women with biopsy-confirmed CIN 2 or CIN 3, not a single hrHPV genotype had a significantly different prevalence by HIV status. The most common hrHPV genotype in women with biopsy-confirmed CIN 2 or CIN 3 was HPV 16, irrespective of either HIV status or age. These results differ slightly from a meta-analysis, which found less HPV 16 in HIV-positive women with cytological high-grade squamous intraepithelial lesions (HSIL) than in the general female population with HSIL (6). Data from sub-Saharan Africa are more limited.

Interestingly and consistent with what has been reported, HPV 16 and 18 contributed to a larger proportion of high-risk HPV types among women with CIN 3 (54%) than among women with CIN 2 (31%). The relative proportion of high-risk HPV infections attributable to HPV 16 and 18 was even greater still in women with cancer (78%). We had only one HIV-positive woman with cancer and therefore can not comment on likely differences in HPV genotype distribution by HIV status among women with cancer in this population. One study of invasive cervical cancers from Kenya and South Africa found a modest increase in HPV 18 among cancers from HIV-positive women, but no significant differences for other hrHPV genotypes (40).

As previously found, both CIN 2 and CIN 3 were much more common in HIV-positive compared to HIV-negative women with a combined prevalence of 9.2% vs. 2.7% in HIV-positive and -negative women, respectively. This increase was observed in all age groups. There remains controversy as to whether or not HIV is associated with an increase in the incidence of cervical cancer (9, 10, 13, 26). Therefore, it is of interest that when we restricted comparisons to hrHPV-positive women; no difference was seen in the prevalence of CIN 3 by HIV status. This finding is consistent with what has been observed in some but not all studies from the U.S. (4, 41, 42). These observations suggest that the impact of HIV-infection on HPV-associated cancers is to increase susceptibility to infection with hrHPV or reactivation of previously acquired hrHPV infection, but that HIV-infection does not necessarily also influence progression rates to CIN 3 or invasive cervical cancer. It is now well-recognized that, although persistent hrHPV infections are a prerequisite for development of CIN 3 and cervical cancer, the pathogenesis of these lesions requires subsequent accumulation of (epi)genetic alterations in the cells that allows them to become immortalized and less sensitive to growth-modulating factors (43).

The performance of cervical cancer screening tests including hrHPV testing in HIV-positive and -negative women has been previously studied (24, 44, 45). The current analysis confirms that sensitivity of hrHPV testing as a screening test is not reduced in HIV-positive women; but, the specificity is decreased. Guidelines from the U.S. currently do not recommend hrHPV testing as screening for HIV-positive women given its low specificity even though health economic models demonstrate that it is an appropriate screening approach (46, 47). Our finding that hrHPV testing maintains a high sensitivity in HIV-positive women supports using HPV testing as a primary screening test even in populations with high HIV prevalence. Nevertheless, there remains a critical research gap to develop additional triage tests, e.g., cancer biomarkers such as p16 immunostaining or Ki67, to identify which hrHPV-positive, HIV-positive women require additional work-up and/or treatment.

A key strength of this study is the rigorous ascertainment of disease. All the colposcopy examinations were carried out by the same team of highly experienced clinicians; and, all cervical biopsies were reviewed by the same experienced gynecological pathologists, providing consistency in the results. An important limitation is the lack of clinical information about HIV disease status, such as HIV viral load, CD4 cell counts, or use of antiretroviral therapy. Because women were recruited from the general community, it is likely that there is an under-representation of women with advanced HIV disease. This may explain the lower hrHPV prevalence and biopsy-confirmed CIN 2 and 3 rates we observed compared to other studies enrolling at HIV care sites. Another limitation was that the HC2 assay did not detect high-risk HPV types in some HC2 positive women. This was most marked in women without disease and some may be explained by false-positive HC2 tests. Genotyping’s sensitivity improved in women with more advanced cervical disease and was slightly better in HIV-positive vs. HIV-negative women.

In conclusion, we have confirmed a high burden of hrHPV infection including multiple type infections and biopsy-confirmed CIN 2 or 3 in HIV-positive women in sub-Saharan Africa. HPV 16 and 35 were consistently the most common high-risk types among HIV-positive and -negative women with or without cervical disease with little differences in the distribution of hrHPV genotypes after stratifying by age and cervical disease status. These findings suggest that screening strategies incorporating hrHPV genotyping and vaccination should be effective in preventing cervical cancer in HIV-positive and -negative women living in sub-Saharan Africa.

## Conflict of Interest Statement

Dr. Lynette Denny has received honoraria for appearing on speaker forums regarding HPV vaccines for GlaxoSmithKline (Cervarix) and MSD/Merck (Gardasil) and has undertaken other studies funded by these companies. Dr. Lynette Denny is not employed by either company, does no consultancy work, has no patents or products in development, and has no financial interest in any way with either company. Dr. Thomas C. Wright Jr. is a consultant to Roche Molecular Diagnostics, BD Diagnostics, GenProbe/Hologic, and Cepheid. Dr. Thomas C. Wright Jr. has received honoraria for appearing on speaker forums regarding HPV diagnostics for Roche Molecular Diagnostics, BD Diagnostics, and GenProbe/Hologic. Dr. Thomas C. Wright Jr. is not employed by any of these companies and has no patents or products in development relevant to this study. Drs. Alicia C. McDonald, Ana I. Tergas, and Louise Kuhn have no competing interests to declare.

## Supplementary Material

The Supplementary Material for this article can be found online at http://www.frontiersin.org/Journal/10.3389/fonc.2014.00048/abstract
