# Supplementary material for: Distribution of Human Papillomavirus Genotypes among HIV-Positive and HIV-Negative Women in Cape Town, South Africa
Source: Front Oncol. 2014 Mar 14;4:48. doi: 10.3389/fonc.2014.00048 (PMC3953716; doi:10.3389/fonc.2014.00048)
Supplement: Supplementary file 1 [file Datasheet.PDF]

Data from Figure 1 – Age-specific HIV, HPV, and advanced cervical disease prevalence in study participants.

Age group	ALL	HPV prevalence (HPV DNA positivity)			CIN 2/CIN 3 prevalence		
		HIV+	HIV−	*p*-Value	HIV+	HIV−	*p*-Value
17–19 years	20.1% (48/239)	75% (36/48)	60.2% (115/191)	0.0575	12.5% (6/48)	1.6% (3/191)	0.0026
20–24 years	24.1% (221/914)	60.6% (134/221)	37.7% (261/693)	<0.0001	5.4% (12/221)	2.7% (19/693)	0.0546
25–29 years	26.9% (243/905)	59.7% (145/243)	23.9% (158/662)	<0.0001	12.8% (31/243)	2.1% (14/662)	<0.0001
30–34 years	20.8% (175/841)	54.9% (96/175)	20.3% (135/666)	<0.0001	15.4% (27/175)	3.6% (24/666)	<0.0001
35–39 years	15.2% (407/2,679)	46.4% (189/407)	19.3% (439/2,272)	<0.0001	8.1% (33/407)	2.9% (65/2,272)	<0.0001
40–44 years	9.5% (147/1,547)	42.2% (62/147)	17.6% (247/1,400)	<0.0001	5.4% (8/147)	3.1% (44/1,400)	0.1412
45–49 years	7.2% (76/1,058)	43.4% (33/76)	13.2% (130/982)	<0.0001	7.9% (6/76)	3.1% (30/982)	0.0250
50–54 years	4.3% (28/645)	53.6% (15/28)	16.5% (102/617)	<0.0001	7.1% (2/28)	2.1% (13/617)	0.1347
55–65 years	4.4% (26/593)	34.6% (9/26)	14.6% (83/567)	0.0059	7.7% (2/26)	1.4% (8/567)	0.0670
All ages	14.6% (1,371/9,421)	52.4% (719/1,371)	20.7% (1,670/8,050)	<0.0001	9.3% (127/1,371)	2.7% (220/8,050)	<0.0001

Data from Figure 2 – Age-specific distribution of specific HR types infections among HIV-positive and HIV-negative women with WNL/CIN 1.

WNL/CIN 1	HIV-positive			HIV-negative			*p*-Value (HIV+ vs. HIV−)		
	**17–29 years**	**30–39 years**	**40–65 years**	**17–29 years**	**30–39 years**	**40–65 years**	**17–29 years**	**30–39 years**	**40–65 years**
Total # of infections	380	283	119	601	445	365	–	–	–
# With HPV 16	36 (9.5%)	29 (10.2%)	13 (10.9%)	57 (9.5%)	54 (12.1%)	58 (15.9%)	0.9956	0.4347	0.1836
# With HPV 18	30 (7.9%)	26 (9.2%)	16 (13.4%)	48 (8.0%)	34 (7.6%)	25 (6.8%)	0.9586	0.4594	0.0248
# With HPV 31	20 (5.3%)	15 (5.3%)	5 (4.2%)	31 (5.2%)	33 (7.4%)	25 (6.8%)	0.9424	0.2622	0.2983
# With HPV 33	22 (5.8%)	10 (3.5%)	10 (8.4%)	43 (7.2%)	23 (5.2%)	16 (4.4%)	0.4023	0.3013	0.0912
# With HPV 35	37 (9.7%)	34 (12.0%)	16 (13.4%)	80 (13.3%)	61 (13.7%)	51 (14.0%)	0.0924	0.5084	0.8850
# With HPV 39	25 (6.6%)	12 (4.2%)	5 (4.2%)	33 (5.5%)	16 (3.6%)	9 (2.5%)	0.4815	0.6592	0.3265
# With HPV 45	25 (6.6%)	28 (9.9%)	16 (13.4%)	44 (7.3%)	50 (11.2%)	31 (8.5%)	0.6579	0.5682	0.1131
# With HPV 51	30 (7.9%)	20 (7.1%)	9 (7.6%)	47 (7.8%)	27 (6.1%)	24 (6.6%)	0.9663	0.5926	0.7105
# With HPV 52	23 (6.1%)	27 (9.5%)	13 (10.9%)	36 (6.0%)	43 (9.7%)	32 (8.6%)	0.9679	0.9565	0.4816
# With HPV 56	21 (5.5%)	18 (6.4%)	2 (1.7%)	35 (5.8%)	20 (4.5%)	16 (4.4%)	0.8450	0.2698	0.2645
# With HPV 58	47 (12.4%)	30 (10.6%)	4 (3.4%)	53 (8.8%)	39 (8.8%)	37 (10.1%)	0.0734	0.4095	0.0219
# With HPV 59	28 (7.4%)	8 (2.8%)	3 (2.5%)	39 (6.5%)	22 (4.9%)	18 (4.9%)	0.5949	0.1613	0.3130
# With HPV 68	36 (9.5%)	26 (9.2%)	7 (5.9%)	55 (9.2%)	23 (5.2%)	23 (6.3%)	0.8654	0.0349	0.8692
# With HPV 16/18	63 (16.6%)	51 (18.0%)	27 (22.7%)	104 (17.3%)	85 (19.1%)	83 (22.7%)	0.7683	0.7155	0.9909

Data from Figure 3 – Age-specific distribution of specific HR types infections among HIV-positive and HIV-negative women with CIN 2/CIN 3.

CIN 2/CIN 3	HIV-positive			HIV-negative			*p*-Value (HIV+ vs. HIV−)		
	17–29 years	30–39 years	40–65 years	17–29 years	30–39 years	40–65 years	17–29 years	30–39 years	40–65 years
Total # of isolates	87	101	21	59	77	90	–	–	–
# With HPV 16	14 (16.1%)	13 (12.9%)	7 (33.3%)	8 (13.6%)	16 (20.8%)	26 (28.9%)	0.6747	0.1569	0.6882
# With HPV 18	3 (3.4%)	10 (9.9%)	0 (0%)	8 (13.6%)	5 (6.5%)	4 (4.4%)	0.0289	0.4175	1.000
# With HPV 31	10 (11.5%)	4 (4.0%)	2 (9.5%)	8 (13.6%)	4 (5.2%)	5 (5.6%)	0.7096	0.7281	0.6150
# With HPV 33	9 (10.3%)	6 (5.9%)	2 (9.5%)	8 (13.6%)	9 (11.7%)	4 (4.4%)	0.5524	0.1714	0.3174
# With HPV 35	10 (11.5%)	16 (15.8%)	4 (19.0%)	9 (15.3%)	16 (20.8%)	18 (20.0%)	0.5076	0.3954	1.000
# With HPV 39	2 (2.3%)	6 (5.9%)	1 (4.8%)	2 (3.4%)	1 (1.3%)	0 (0%)	1.000	0.1416	0.1892
# With HPV 45	2 (2.3%)	4 (4.0%)	3 (14.3%)	1 (1.7%)	5 (6.5%)	13 (14.4%)	1.000	0.5033	1.000
# With HPV 51	4 (4.6%)	7 (6.9%)	0 (0%)	1 (1.7%)	3 (3.9%)	2 (2.2%)	0.6484	0.5178	1.000
# With HPV 52	3 (3.4%)	7 (6.9%)	1 (4.8%)	3 (5.1%)	7 (9.1%)	5 (5.6%)	0.6858	0.7798	1.000
# With HPV 56	7 (8.0%)	3 (3.0%)	0 (0%)	0 (0%)	3 (3.9%)	1 (1.1%)	0.0417	1.000	1.000
# With HPV 58	13 (14.9%)	13 (12.9%)	1 (4.8%)	7 (11.9%)	7 (9.1%)	8 (8.9%)	0.5955	0.4288	1.000
# With HPV 59	2 (2.3%)	4 (4.0%)	0 (0%)	1 (1.7%)	0 (0%)	2 (2.2%)	1.000	0.1345	1.000
# With HPV 68	8 (9.2%)	8 (7.9%)	0 (0%)	3 (5.1%)	1 (1.3%)	2 (2.2%)	0.5259	0.0797	1.000
# With HPV 16/18	16 (18.4%)	23 (22.8%)	7 (33.3%)	15 (25.4%)	21 (27.3%)	28 (31.1%)	0.3079	0.4904	0.8436
